# Characteristics of the Delayed or Refusal Therapy in Breast Cancer Patients: A Longitudinal Population-Based Study in Taiwan

**DOI:** 10.1371/journal.pone.0131305

**Published:** 2015-06-26

**Authors:** Su Jing Chen, Pei-Tseng Kung, Kuang Hua Huang, Yueh-Hsin Wang, Wen-Chen Tsai

**Affiliations:** 1 Department of Health Services Administration, China Medical University, Taichung, Taiwan; 2 Department of Pharmacy, China Medical University Hospital, Taichung, Taiwan; 3 Department of Public Health, China Medical University, Taichung, Taiwan; 4 Department of Healthcare Administration, Asia University, Taichung, Taiwan; Taipei Medical University, TAIWAN

## Abstract

**Background:**

The evidence indicated breast cancer was a cancer with high survival rate. However, there were still some breast cancer patients delaying or refusing therapy. So we conducted a cohort study to explore the relationship between characteristics of breast cancer patients and delay or refusal of therapy within four months after cancer diagnosed.

**Methods:**

This was a retrospective national population-based study from 2004 to 2010 in Taiwan. This study included 35,095 patients with new diagnosis breast cancer from Taiwan Cancer Registry Database. Several analysis methods, including t test, Chi-square test, generalized estimating equations of logistic regression analysis, and Cox proportional hazards model, were performed to explore the characteristics of these patients and the relative risk of mortality with delay or refusal of therapy.

**Results:**

Our study showed that the overall survival rates were significantly different (*p* <0.05) between the breast cancer patients who delayed or refused therapy and those with treatment. The patients who delayed or refused therapy had lower 5-year overall survival rate compared with the treated group. The related factors included age, Charlson comorbidity index, cancer staging (OR = 1.30–19.69; *p* <0.05), other catastrophic illnesses or injuries and the level of diagnostic hospitals. However, the patients with different income levels and degree of urbanization in living area were not statistically significant factors.

**Conclusion:**

Our results demonstrated that age and cancer staging were the main patient characteristics affecting whether the patients delayed or refused therapy. The delay or refusal of treatment was associated with the level of diagnosing hospital.

## Introduction

Early diagnosis and timing treatment can improve the survival and the quality of life for the cancer patients[1]. According to the projection by the International Agency for Research on Cancer (IARC), the global cancer burdens will increase to nearly double by 2030 [2] and, the Asian population will account for 60% proportionally [3]. The annual reports of in cancer statistics by American Cancer Society (ACS) in 2013, showed that the mortality rate of female due to cancer decreased annually by 1.5% from 2005 to 2009[4]. The overall 5-year relative survival rates of breast cancer patients were 89% in US [5], 85% in UK [6], and 86.5% in Taiwan [7]. The data indicated breast cancer was a cancer with high incidence and high survival rate for women in comparison to other cancers.

The ACS statistics showed that, among the women with invasive breast carcinoma, about 1% of the patients in early stage (stage I or II) refused any treatment, and 7% of those in late stage (stage III or IV) did not receive any treatment[8]. Early cancer treatment resulted in higher survival rate although some studies have shown that higher proportion of breast cancer patients in early-stage without insurance did not receive medical treatment compared to those with insurance due to financial factors [9]. The burden of cancer is different among ethnic groups in the United States. It was reported that the African American had significantly higher mortality by breast cancer compared to the Caucasian patients[10]. In general, variations of the health care system in different countries have derived a variety of scenarios leading to delay or refusal of therapy [11].

Medical treatments of breast cancer include surgery, chemotherapy, radiotherapy and hormone therapy. In addition to classical therapies, alternative or complementary therapies are also available for cancer patients. Previous studies have shown that patients may need to face many physical and psychological adaption after diagnosed with cancer, including uncertainty of disease progression and fear of death [12–14], change of body image [15, 16], lack of effective doctor-patient communication and information [17, 18], intolerable adverse effects of chemotherapy such as emesis and nausea, and deterioration in quality of life [19, 20]. These physical and psychological challenges and patients’ assessment of effectiveness and risk for cancer therapy [21, 22] may result in delay of medical treatment.

The National Health Insurance (NHI) has been implemented since 1995 in Taiwan, and the coverage rate has reached 99% by the end of 2004[23]. Exemption from copayment on cancer therapy reduced the financial burden and increased the accessibility of healthcare for cancer patients [24–28]. Although the healthcare models for cancer treatment and financial burdens of cancer patients are varied in different countries, there are still breast cancer patients delaying or refusing therapy when cancer care is almost 100% accessible for patients in Taiwan. The aim of this study is to explore the characteristics of breast cancer patients who delayed or refused treatment in Taiwan. Our finding might point out some problems for cancer therapy in different health care systems, and could serve as a reference to reduce the number of patients delaying or refusing treatments and to increase survival rate.

## Methods

### Data source

We conducted a retrospective national population-based study from 2004 to 2010. This databases including the National Health Insurance Research Database (NHIRD), Taiwan Cancer Registry Database (CRD) and the Cause of Death Database. This study protocol was approved by the institutional review board of the China Medical University and Hospital, Taiwan (IRB No: CMUH102-REC3-076). All patient identification information has been deleted prior to analysis, and personal privacy was under protection from using these data.

### Study population

There were 45,911 patients of newly diagnosed breast cancer in Taiwan from 2004 to 2010 [29]. This study included 35,095 patients with cancer new diagnosis code C500-C509 (ICD9 code: 174–175, 217) from Taiwan Cancer Registry Database, thus the sampling has covered over 76.4% of the national breast cancer population. The patients were followed-up till December 31, 2012.

### Study variables and measurements

The Cancer Registry Database in the Health Promotion Administration defines the aggressive treatment as treatment within 120 days of diagnosis. Since the staging might be different after 120 days after diagnosis, treatment of cancer with different stages does not belong to aggressive treatment anymore. Another study analyzing the cumulative treatment rate in one year showed that there was only slight increase in the proportion of patients who started treatment after four months of new diagnosis [30]. Consequently, this study defined “delay or refuse therapy” as no classical therapies including surgery, radiotherapy, chemotherapy, hormone therapy, CCRT (concurrent chemoradiotherapy), targeted therapy, excluding palliative therapy, within four months following confirmed diagnosis of breast cancer.

Demographic variable was the age at cancer diagnosis. The comorbidity was based on the Charlson comorbidity index (CCI), and was defined by the injuries or diseases, except cancers, prior to the first cancer diagnosis. Severity of cancer was differentiated by four stages. The analysis variables defining socioeconomic status included the monthly salary and insurance status (employees / employers, farmers or fishers, low-income household, unemployed / retired / others). Definition of urbanization leveled from 1 to 7, with the level 1as the highest and level 7 as the lowest. The level of diagnosing hospital was divided into medical center, regional hospital, district hospital and primary care clinic. Moreover, the hospital ownership included public and private.

### Statistical analysis

The descriptive statistics was performed on the number of patients and the percentage of patients who delayed or refused treatment between 2004 and 2010. The characteristics of patients analyzed included age, degree of urbanization, socioeconomic status (monthly salary, low-income household), health status (comorbidity, CCI, staging), and the level of diagnosing hospital.

The demographic and the ratio of the number of breast cancer patients who delayed or refused therapy were analyzed with t test and Chi-square test, respectively, to see if there was difference between patients who received treatment or not.

The logistic regression involving generalized estimating equation (GEE) was performed to evaluate variables related to the risk of delaying or refusing therapy without the potential bias from cluster effects derived from the effects of treatment at the same hospitals. Finally, the Cox proportional hazards model in survival analysis was conducted with adjustment of covariates, including basic characteristics, socioeconomic status, health status and the level of diagnosing hospital, to analyze the variables related to the survival of the patients who delayed or refused therapy. SAS version 9.2 was used in all analyses.

## Results

**Fig 1:**
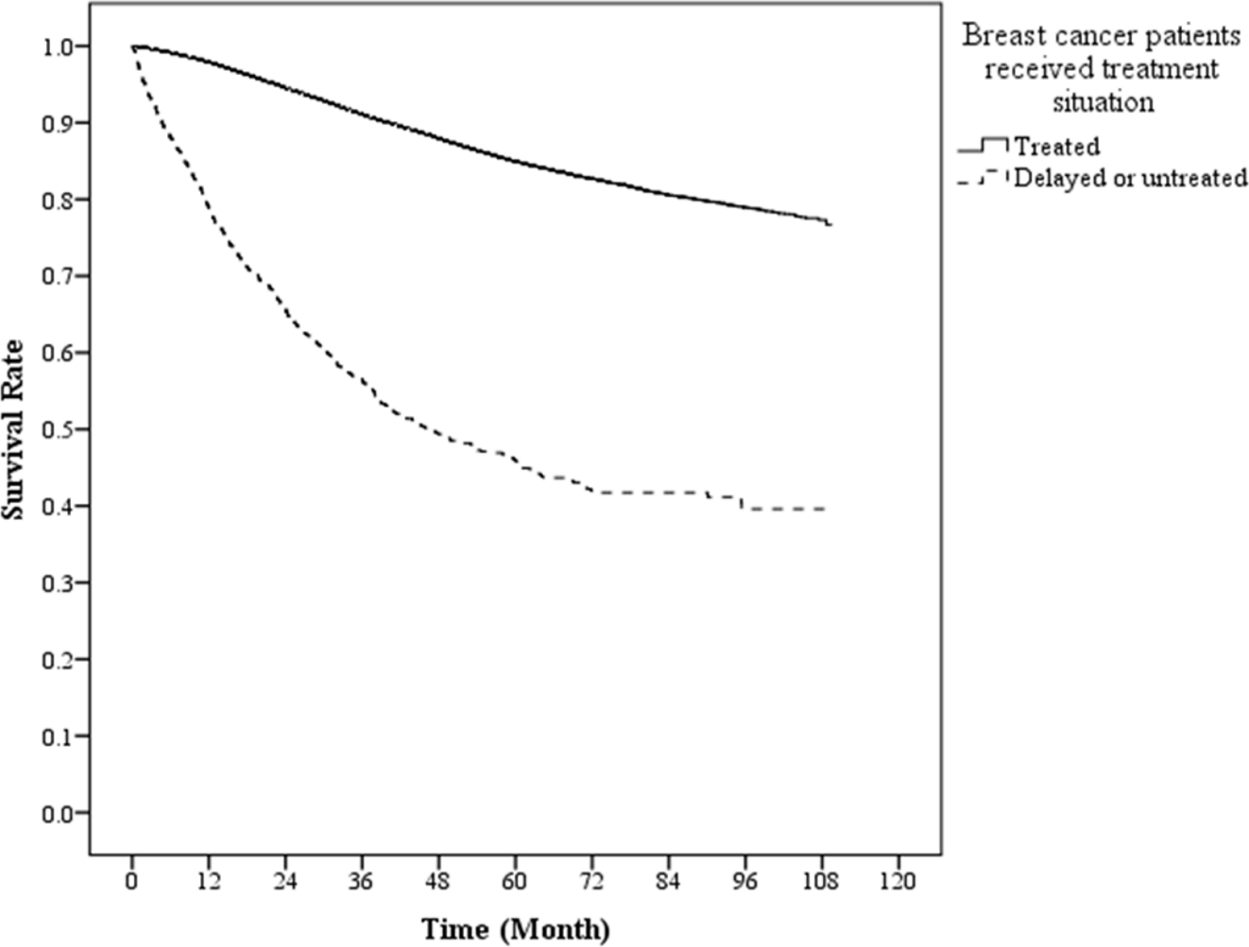
Overall survival curves between treated and delayed/untreated patients of breast cancer.

**Fig 2:**
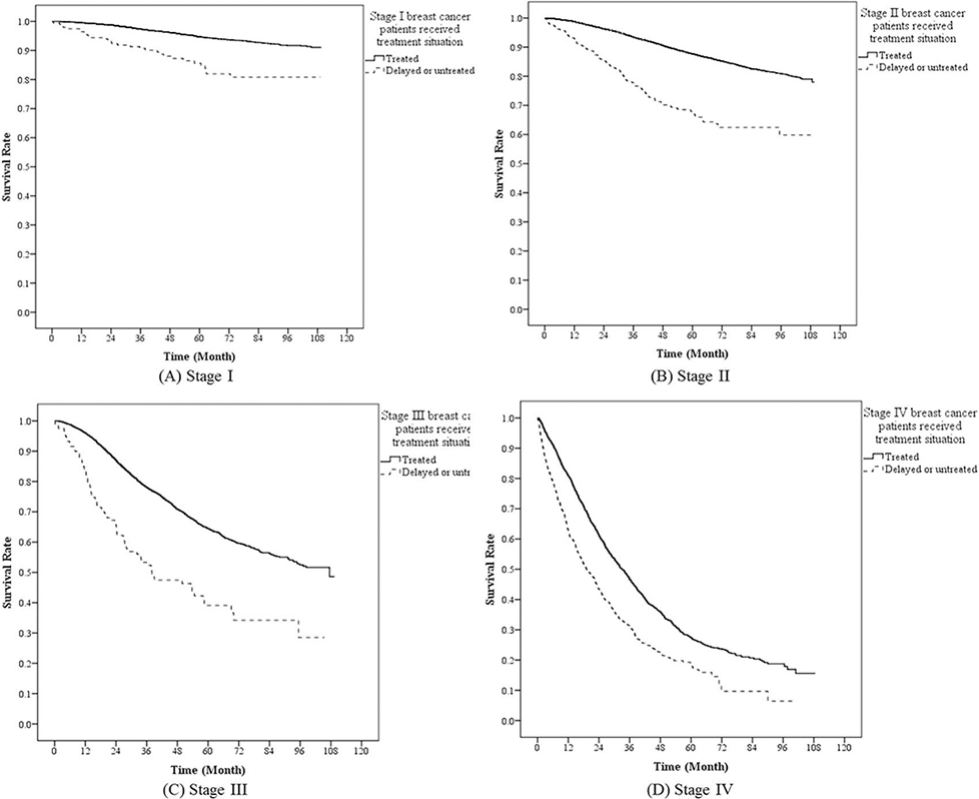
Survival curves between treated and delayed/untreated patients of breast cancer at different stages.

This study showed that there was significant difference (*p* <0.05) in overall survival between patients who delayed or refused therapy and those with treatment (Fig 1). The 5-year survival rate was 85% and 45% for patients with treatment and those who delayed or refused therapy, respectively. We also displayed the survival situation at different stages between treated patients and delayed/refused treatment patients in Fig 2. The survival difference between two groups was more significant at stage II and stage III.

Table 1 showed the higher proportion of older patients (age ≥ 75 years old, 6.91%) delayed in treatment or remained untreated. In terms of socioeconomic status, more patients with lower salary (≤ 17280, 4.76%) and as the insured dependents (3.92%) belonged to the group who delayed or refused therapy. Higher percentage of patients with status of low-income household (6.01%), unemployed, and retired (4.90%) were in the group who delayed or refused therapy. Patients with higher comorbidity index (CCI≧7, 8.24%) were more likely to delay or refuse therapy. In terms of cancer severity, significantly higher percentage of end-stage cancer (stage IV, 25.85%) patients delayed or refused therapy. On the other hand, patients diagnosed at the regional hospitals and medical center had the highest and lowest percentage to delay or refuse therapy, respectively (5.13% vs 2.65%).

**Table 1:** Bivariate analysis for the treatment choice of the characteristics in breast cancer patients.

Variables		Total		Delay or untreated		Treated		
		N	%	N	%	N	%	P value
**Total**		35095	100.00	1243	3.54	33852	96.46	
**Age at diagnosed**								<0.001
	≤ 44	8736	24.89	301	3.45	8435	96.55	
	45–54	12865	36.66	406	3.16	12459	96.84	
	55–64	7889	22.48	257	3.26	7632	96.74	
	65–74	3854	10.98	158	4.10	3696	95.90	
	≥75	1751	4.99	121	6.91	1630	93.09	
**Mean age at diagnosed**		52.53	11.75	54.48	13.41	52.45	11.68	<0.001
**Monthly salary**								<0.001
	≤ 17280	6033	17.19	287	4.76	5746	95.24	
	Insured dependent	7935	22.61	311	3.92	7624	96.08	
	17281–22800	9958	28.37	335	3.36	9623	96.64	
	≥ 22801	11169	31.83	310	2.78	10859	97.22	
**Insurance Status**								<0.001
	Employees/employers	24374	69.45	761	3.12	23613	96.88	
	Farmers or fishers	4377	12.47	168	3.84	4209	96.16	
	Low-income household	283	0.81	17	6.01	266	93.99	
	Unemployed, retired, others	6061	17.27	297	4.90	5764	95.10	
**Urbanization level of residence location**								0.495
	Level 1	12948	36.89	443	3.42	12505	96.58	
	Level 2	11289	32.17	408	3.61	10881	96.39	
	Level 3	4391	12.51	165	3.76	4226	96.24	
	Level 4	3998	11.39	133	3.33	3865	96.67	
	Level 5	493	1.40	23	4.67	470	95.33	
	Level 6	882	2.51	37	4.20	845	95.80	
	Level 7	1094	3.12	34	3.11	1060	96.89	
**Level of diagnosing hospital**								<0.001
	Medical center	20996	59.83	557	2.65	20439	97.35	
	Regional hospital	9827	28.00	504	5.13	9323	94.87	
	District hospital	1893	5.39	92	4.86	1801	95.14	
	Primary medical clinic	2379	6.78	90	3.78	2289	96.22	
**Hospital ownership**								0.366
	Public	14290	40.72	522	3.65	13768	96.35	
	Private	20805	59.28	721	3.47	20084	96.53	
**Charlson comorbidity index**								<0.001
	0–3	29673	84.55	845	2.85	28828	97.15	
	4–6	3419	9.74	233	6.81	3186	93.19	
	≧ 7	2003	5.71	165	8.24	1838	91.76	
**Other catastrophic illnesses or injuries**								<0.001
	without	33981	96.83	1174	3.45	32807	96.55	
	with	1114	3.17	69	6.19	1045	93.81	
**Staging**								<0.001
	Stage I	12226	34.84	196	1.60	12030	98.40	
	Stage II	17185	48.97	364	2.12	16821	97.88	
	Stage III	3502	9.98	119	3.40	3383	96.60	
	Stage IV	2182	6.22	564	25.85	1618	74.15	

Table 2 showed the relative risks of the breast cancer characteristics on delaying or refusing therapy with GEE logistic regression analysis. The older groups had higher risk of delaying or refusing therapy, especially in patients aged ≧75 years (OR = 1.40, 95% CI:1.11–1.79; *p* < 0.05). Moreover, patients with other catastrophic illnesses in addition to breast cancer were easier to delay or refuse therapy (OR = 1.58, 95% CI: 1.16–2.16; P <0.05). With the CCI to define the severity of comorbidity, the group with higher CCI 4–6 was more likely to delay or refuse therapy (OR = 1.3, 95% CI: 1.13–1.48; *p* < 0.05). In terms of cancer staging, significantly more patients with more advanced cancer delayed or refused therapy comparing to the group of early (stage I) cancer (OR = 1.30–19.69; *p* < 0.05). Respecting the level of diagnosing hospital, more patients at the lower hospital level delayed or refused therapy compared to those at the medical centers (OR = 1.52–2.08; P <0.05).

**Table 2:** Analysis of logistic regression model with generalized estimating equations for the correlations between patient choice and patient characteristics in breast cancer patients.

Variables	OR	95% CI		P value
**Age at diagnosed**				
≦ 44 (reference)				
45–54	0.83	0.70	0.97	0.023
55–64	0.73	0.63	0.85	<0.001
65–74	0.88	0.70	1.09	0.252
≧ 75	1.40	1.11	1.79	0.006
**Monthly salary (NTD)**				
≤ 17280(reference)				
Insured dependent	0.84	0.70	1.01	0.070
17281–22800	0.82	0.66	1.02	0.079
≧ 22801	0.83	0.64	1.05	0.120
**Insurance Status**				
Employees/employers (reference)				
Farmers or fishers	1.22	0.98	1.54	0.080
Low-income household	0.96	0.56	1.67	0.900
Unemployed, retired, others	1.13	0.94	1.36	0.190
**Urbanization level of residence location**				
Level 1 (reference)				
Level 2	0.95	0.82	1.11	0.494
Level 3	0.93	0.74	1.19	0.584
Level 4	0.77	0.60	1.00	0.052
Level 5	0.99	0.63	1.55	0.971
Level 6	0.83	0.61	1.14	0.249
Level 7	0.71	0.49	1.03	0.073
**Level of diagnosing hospital**				
Medical center (reference)				
Regional hospital	2.08	1.49	2.86	<0.001
District hospital	1.77	1.28	2.46	0.001
Primary medical clinic	1.52	1.06	2.16	0.021
**Hospital ownership**				
Public (reference)				
Private	0.94	0.68	1.30	0.716
**CCI score**				
0–3 (reference)				
4–6	1.30	1.13	1.48	<0.001
Above 7	1.14	0.94	1.39	0.181
**Other catastrophic illnesses or injuries**				
without (reference)				
with	1.58	1.16	2.16	0.004
**Staging**				
Stage I (reference)				
Stage II	1.30	1.11	1.54	0.002
Stage III	1.95	1.55	2.44	<0.001
Stage IV	19.69	14.59	26.58	<0.001

Note: Event: delay or refusal of treatment

Table 3 demonstrated the effects of delaying or refusing therapy on survival of breast cancer patients. Overall, the patients who delayed or refused therapy had significantly higher risk of mortality (HR = 1.67, 95% CI: 1.53–1.82; *p* < 0 .05).

**Table 3:** Effect of delayed or untreated versus treated patients on survival in breast cancer.

Variables				Delay or untreated				Treated				Adj.			
		Total		Alive		Death		Alive		Death		HR*	95%	CI	P value
		N	%	N	%	N	%	N	%	N	%				
**Total**		35095	100.00	587	1.67	656	1.87	28833	82.16	5019	14.30	1.67	1.53	1.82	<0.001
**Age at diagnosis(years)**															
	≦ 44	8736	24.89	193	2.21	108	1.24	7416	84.89	1019	11.66	1.34	1.09	1.66	0.006
	45–54	12865	36.66	211	1.64	195	1.52	10912	84.82	1547	12.02	1.71	1.45	2.00	<0.001
	55–64	7889	22.48	104	1.32	153	1.94	6501	82.41	1131	14.34	1.69	1.41	2.03	<0.001
	65–74	3854	10.98	53	1.38	105	2.72	2948	76.49	748	19.41	1.90	1.52	2.38	<0.001
	≧ 75	1751	4.99	26	1.48	95	5.43	1056	60.31	574	32.78	2.36	1.81	3.06	<0.001
**Monthly salary(NTD)**															
	≦ 17280	6033	17.19	139	2.30	148	2.45	4677	77.52	1069	17.72	1.70	1.41	2.05	<0.001
	Insured dependent	7935	22.61	107	1.35	204	2.57	6378	80.38	1246	15.70	1.89	1.60	2.23	<0.001
	17281–22800	9958	28.37	146	1.47	189	1.90	8054	80.88	1569	15.76	1.84	1.56	2.16	<0.001
	≧ 22801	11169	31.83	195	1.75	115	1.03	9724	87.06	1135	10.16	1.35	1.10	1.66	0.004
**Insurance Status**															
	Employees/employers	24374	69.45	396	1.62	365	1.50	20536	84.25	3077	12.62	1.61	1.43	1.81	<0.001
	Farmers or fishers	4377	12.47	55	1.26	113	2.58	3424	78.23	785	17.93	1.80	1.44	2.25	<0.001
	Low-income household	283	0.81	7	2.47	10	3.53	199	70.32	67	23.67	2.98	1.24	7.18	0.015
	Unemployed, retired, others	6061	17.27	129	2.13	168	2.77	4674	77.12	1090	17.98	1.71	1.43	2.05	<0.001
**Urbanization level of residence location**															
	Level 1	12948	36.89	229	1.77	214	1.65	10816	83.53	1689	13.04	1.52	1.31	1.78	<0.001
	Level 2	11289	32.17	192	1.70	216	1.91	9295	82.34	1586	14.05	2.01	1.72	2.35	<0.001
	Level 3	4391	12.51	61	1.39	104	2.37	3550	80.85	676	15.40	1.70	1.35	2.14	<0.001
	Level 4	3998	11.39	66	1.65	67	1.68	3215	80.42	650	16.26	1.49	1.13	1.95	0.004
	Level 5	493	1.4	10	2.03	13	2.64	385	78.09	85	17.24	2.17	1.06	4.42	0.034
	Level 6	882	2.51	12	1.36	25	2.83	686	77.78	159	18.03	2.78	1.72	4.50	<0.001
	Level 7	1094	3.12	17	1.55	17	1.55	886	80.99	174	15.90	0.89	0.49	1.63	0.713
**Level of diagnosing hospital**															
	Medical center	20996	59.83	236	1.12	321	1.53	17340	82.59	3099	14.76	1.67	1.48	1.89	<0.001
	Regional hospital	9827	28	268	2.73	236	2.40	8025	81.66	1298	13.21	1.57	1.35	1.82	<0.001
	District hospital	1893	5.39	43	2.27	49	2.59	1540	81.35	261	13.79	1.76	1.22	2.54	0.003
	Primary medical clinic	2379	6.78	40	1.68	50	2.10	1928	81.04	361	15.17	1.97	1.41	2.75	<0.001
**Hospital ownership**															
	Public	14290	40.72	265	1.85	257	1.80	11780	82.44	1988	13.91	1.68	1.47	1.93	<0.001
	Private	20805	59.28	322	1.55	399	1.92	17053	81.97	3031	14.57	1.67	1.49	1.88	<0.001
**Charlson comorbidity index**															
	0–3	23247	66.24	351	1.51	41	0.18	22508	96.82	347	1.49	4.30	3.02	6.11	<0.001
	4–6	5132	14.62	117	2.28	198	3.86	3498	68.16	1319	25.70	1.80	1.53	2.12	<0.001
	≧7	6716	19.14	119	1.77	417	6.21	2827	42.09	3353	49.93	1.55	1.39	1.72	<0.001
**Other catastrophic illnesses or injuries**															
	without	33385	95.13	564	1.69	584	1.75	27723	83.04	4514	13.52	1.62	1.48	1.78	<0.001
	with	1710	4.87	23	1.35	72	4.21	1110	64.91	505	29.53	2.34	1.75	3.13	<0.001
**Staging**															
	Stage I	12226	34.84	166	1.36	30	0.25	11408	93.31	622	5.09	2.45	1.68	3.58	<0.001
	Stage II	17185	48.97	246	1.43	118	0.69	14706	85.57	2115	12.31	1.91	1.58	2.30	<0.001
	Stage III	3502	9.98	49	1.40	70	2.00	2222	63.45	1161	33.15	1.83	1.43	2.34	<0.001
	Stage IV	2182	6.22	126	5.77	438	20.07	497	22.78	1121	51.37	1.51	1.35	1.69	<0.001

Note: * Treated patients as the references group

In Table 3 we further conducted stratified analysis between treated and delayed/refused groups for each variable. The higher relative risk in mortality (HR = 1.34–2.36, 95% CI: 1.09–3.06; p < 0 .05) between treated and delayed/refused group increased with the age increasing. The relative risk of mortality between treated and delayed/refused groups was higher for some groups such as the low-income household (HR = 2.98, 95% CI: 1.24–7.18), patients with stage I (HR = 2.45, 95% CI: 1.68–3.58), patients with comorbidity severity CCI 0–3 (HR = 4.30, 95% CI: 3.02–6.11), and patients with the presence of other catastrophic illness (HR = 2.34, 95% CI: 1.75 to 3.13).

## Discussion

This study analyzed the characteristics of breast cancer patients and found that delay or refusal of therapy was related to age, diagnosing hospital, comorbidity severity, other catastrophic illnesses and staging. This study showed that proportion of patients delaying or refusing therapy increased with the age increasing, especially for the group aged ≧75. Previous study showed that the risk of delaying or refusing therapy was higher for the younger breast cancer patients than the older ones was also found in the Carolina Breast Cancer Study (CBCS) [31]. As our results, the study found that a higher proportion of older patients delayed or refused therapy, potentially due to poor health. This is in consistent with earlier reports showing a higher proportion of older cancer patients refused treatments [32, 33] with major reasons such as health function status, comorbidity status, cultural and emotional factors to give up aggressive treatment to prevent accompanying pain since the old age, [34], and partially because of living alone or disability[35]. Furthermore, the average age of diagnosis was 52 in this study compared to 60 for the breast cancer patients of the US [5]. According to previous studies, younger age of diagnosis for breast cancer patients was gene expression related. Without timing treatment, the outcome could be worse [36]. Sariegoet et al. found that of breast cancer patients with <40 years of age had a higher proportion of advanced disease with stage III or IV [37]. Gabriel and Domchekalso also found that the patients diagnosed at age of 35–40 years old with advanced stages had poorer prognosis [38]. On the other hand, older patients had well differentiated tumors and responded better to pharmacotherapies [39]. However, our study showed the higher percentages (33.22–55.25%) of late stage (stage IV) breast cancer patients in all age subgroups, especially in the age 55–64 group (55.25%, table not shown). The overall survival outcome for breast cancer patients can therefore be improved if the proportion of patients with older ages who delay or refuse therapy is reduced.

This study demonstrated the socioeconomic status such as low-income and different salary did not have influences on delay or refusal of therapy. Therefore, it is obvious that socioeconomic status is not the main reason of delay or refusal of therapy for Taiwanese breast cancer patients. According to previous studies on other countries, the minority (Hispanic and African American) were more likely to delay or refuse therapy, and had higher relative risk of mortality [10, 40], because of lack of insurance or low socioeconomic status [40]. The economic burden of medical care of cancer is usually the point of impact on delay or refusal of therapy for cancer patients [3, 41]. The differences in reasons for delay or refusal of therapy may stem from the diversity in healthcare policies in different countries. In the Taiwanese National Health Insurance system, the minority cancer patients such as the group of low income were provided with subsidy such waiver of deductibles to guarantee the accessibility of medical care and the rights. In addition, all cancer patients were exempted from copayment for cancer treatments in Taiwan National Health Insurance system. The diversities of healthcare system and polices between countries may be the reason why findings regarding to delay and refuse therapy by breast cancer patients in the current study are significantly different from earlier researches. The overall survival of breast cancer patients following treatment is thus affected [10, 11, 42].

This study found that regarding to cancer severity, the ratio of delay or refusal of therapy was higher for more advanced breast cancer patients. In consistent with earlier study (delay of therapy for ≧60 days), the risk of mortality by delay of therapy was higher for patients with advanced cancer than those at early stage [42]. In terms of comorbidity by Charlson comorbidity index (CCI), the ratio of delay or refusal of therapy was also higher for the patients with CCI≧7. Previous studies also showed that patients with terminal cancers were more likely to refuse therapy, furthermore, some were even reluctant to accept further examination to get a definite diagnosis with cancer staging. Therefore, some patients who delayed or refused therapy did not have cancer staging confirmed [43].

Delay therapy can be divided into three phases: primary delay (patient delay: from the onset of symptoms to visit the doctor), secondary delay (from the first visit to the confirmed diagnosis), and tertiary delay (from the diagnosis to start therapy) [36]. Among them, the clinician delay belongs to secondary delay and tertiary delay. Delay in any phase or no treatment would cause cancer progression to advanced stages, and significantly affects the treatment outcome [44, 45]. Phases and characteristics of delay or refusal of therapy are different among different cancers and stages. For breast cancer, the major type of delay or no treatment was tertiary delay, and followed by primary delay[45]. Furthermore, phases of therapy delay also depend on region and country (cancer care system), patient characteristics, age as well as cancer staging. Study by Wagner et al. has shown that breast cancer patients delayed surgeries after diagnosis in relation to types of surgeries. There were more delay of surgeries for patients with total mastectomy than those with breast-conserving surgery and reconstructive surgery [33]. Moreover, Jassem et al. showed that the total delay time was about 14.4 (range: 11.5–29.4) weeks in breast cancer patients from 12 different countries surveyed; the duration of primary delay (patient delay) was 4.7 (range: 3.4–6.2) weeks. Longer patient delay happened for patients who distrusted and disregarded medicine. The patients who took routine self-examination, were fear, had higher education, were employed and lived in high urbanization area had shorter delay in therapy [11].The current study focused on the tertiary delay that was at least 120 days between diagnosis and start of therapy. It was also found that patients with employment had lower ratio to delay or refuse therapy. However, our data did not show differences in proportion of delay or refusal of therapy between urbanization levels of residential areas. The study conducted by Tsai et al. collected 109 valid semi-structured questionnaire to investigate the reasons of untreated or interrupted treatment within 4 months among oral, colon, breast and cervical cancer patients [30]. The major factors of refusing treatment in breast cancer patients including the fear of surgery, poor response to therapy and poor life quality following therapy accounted for 33.33%; fear of adverse effects of chemotherapy or radiotherapy, economic burden of household or busy job, and feeling guilty accounted for 22.22%; fear of outlook change following therapy, increased family burden, and fear of others’ knowing of disease accounted for 11.11%[30]. In clinical observation, physical suffering was the most primary care in advanced cancer patients, and almost needed to use the sedation to relieve patients' suffering. However, the psychological distress existed in the whole care process. Citrin et al. reported that the reason to delay or refuse therapy was the patients’ own perception and belief to refuse therapy and turned to alternative therapies [46]. Citrin et al. reported cancer patients may refuse therapy due to the attitude of physicians (such as indifferent, callousness and unnecessary harshness), fear of side effects of treatment and trust in alternative therapies (such as taking fresh fruits, vegetables and nutritional supplements) [46]. Additionally, previous studies also showed that the causes of refusal therapy included patient health status, accessibility of disease information, optimism to the disease, and the interaction with the medical staff. The encouragement from the medical staff was [47] important support for the cancer patients to face the treatments.

For the influence of tertiary delay, Smith et al. found that breast cancer patients at 15–39 years of age (young age) with treatment delay time of > 6 weeks had lower 5-year survival rate compared to those with delay of <2 weeks (*p* = 0.03) [40]. In addition, study by McLaughlin et al. showed that within the group of breast cancer patients with mean age of 61.6 and low income, the patients delayed treatment for ≧ 60 days had worse overall survival than those delayed for < 60 days (*p* = 0 .05)[42]. The current study showed that delay or refusal of treatment for at least 120 days led to higher risk of mortality (*p* < 0 .05), which is consistent with results of previous studies.

Moreover, the diagnosing hospital is also one factor for the patients to delay or refuse therapy. We found that the ratio of patients who delayed or refused therapy was lower for private hospitals or medical centers. It might be due to the trust of patients to the hospitals, or different administrative systems between hospitals.

This study is a retrospective analysis on secondary databases. Some relevant factors such as patient occupation, marital status, lifestyle, medical knowledge, health behaviors, physical and psychological status, and family care and support were not able to be incorporated.

## Conclusion

It is an urgent issue to face the growing economic burden of cancer care all over the world. Delay or refusal of therapy also has impacts on medical burden. The breast cancer patients may delay or refuse therapy due to lack of motivation for treatments by personal preferences, and lead to worse health outcome and lower overall survival. In this study, we found that age and cancer staging were the main factors for delay or refusal of therapy for breast cancer patients. From medical point of view, it is worthwhile to achieve patient-centered care by well communication with patients on the treatment plan, potential obstacles and available support and resources during treatment, such as side effects of treatment, financial support, psychological support from social workers and hospice. The reasonable treatment strategies may thus be developed together with the patients, such that the patients feel compassionate and nonjudgmental attitude from the medical staff. The proportion of patients with delay or refusal of therapy can be decreased and improve the healthcare quality for breast cancer patients.
